# 活性氧刺激响应纳米载体

**DOI:** 10.3724/SP.J.1123.2020.11014

**Published:** 2021-02-08

**Authors:** Wen ZHOU, Kaiguang YANG, Baofeng ZHAO, Lihua ZHANG, Yukui ZHANG

**Affiliations:** 1.中国科学院大连化学物理研究所, 中国科学院分离分析化学重点实验室, 辽宁 大连 116023; 1. CAS Key Laboratory of Separation Science for Analytical Chemistry, Dalian 116023, China; 2.中国科学院大学, 北京 100049; 2. University of Chinese Academy of Science, Beijing 100049, China

**Keywords:** 活性氧, 刺激响应, 纳米载体, 纳米颗粒, reactive oxygen species (ROS), stimuli-responsive, nanocarriers, nanoparticles (NPs)

## Abstract

纳米载体一般是由天然高分子或人工合成高分子组成的、纳米级范畴的运输系统,具有减少药物毒性、提高药物的靶向性、增加药物有效性等优点。随着生物医学技术的进步,有研究表明,作为氧化代谢产物的活性氧(ROS)在疾病部位常常伴随着过表达的异常现象。基于此,近年来ROS刺激响应纳米载体获得了关注和发展,以不同响应机制的ROS响应基团为基础,发展了一系列的ROS响应纳米载体,实现了疾病部位ROS刺激下的药物特异性可控释放。该文聚焦于近年来常用于纳米载体的ROS响应基团,依据元素划分为两大类:硫族元素类响应基团(硫醚、缩硫酮、硒化物、二硒化物、碲化物)和其他元素类响应基团(芳香硼酸酯、过氧草酸酯、二茂铁);通过不同的设计理念将其引入纳米载体,根据ROS响应纳米载体的不同响应机制(疏水-亲水相变、断裂),探讨了载体各自的ROS响应情况、体外药物释放情况,以及在活体中的应用情况。

## Abstract (en)

Nanocarriers are nanoscale delivery systems composed of natural or synthetic polymers, which are advantageous in reducing drug toxicity while improving drug targeting and utilization. With the advancement of biomedical technology, it is revealed that reactive oxygen species (ROS), a class of oxidative metabolites, show abnormal overexpression in disease-related parts of the body. Hence, ROS stimuli-responsive nanocarriers have gained increasing attention, and recent developments are expected to realize controllable drug release. Based on linkers with different ROS-responsive mechanisms, a series of ROS-responsive nanocarriers have been designed to achieve specific controlled drug release under the stimulation of the ROS at the disease site. This article mainly focuses on ROS-responsive linkers, which have been commonly used for the synthesis of nanocarriers in recent years. Accordingly, the linkers are classified as chalcogen-containing responsive linkers (thioether, thioketal, selenide, diselenide, and telluride) and responsive linkers containing other elements (arylboronic ester, ferrocene, and peroxalate ester). ROS stimuli-responsive nanocarriers are fabricated by introducing ROS-responsive linkers in different design principles. Owing to the ROS-responsive linkers, the nanocarriers follow different responsive mechanisms, including hydrophobic-to-hydrophilic phase transition and cleavage. This article discusses the degree of responsiveness of nanocarri-ers and the specific release of drugs from nanocarriers upon ROS-stimuli, as well as their applications in vivo. In particular, on the basis of intelligent drug release and precision medicine, this article also emphasizes the importance of the biocompatibility and biodegradability of nanocarriers.

功能材料近年来发展蓬勃,而且应用领域广泛。相小超等^[1]^总结了应用于蛋白质组研究的功能材料,如磁性纳米材料、金属有机骨架材料等,该类功能材料能够克服传统蛋白质组学方法灵敏度低、准确性差等缺点。除此之外,它在生物医药领域、功能材料中也有着广泛的研究。

在生物医药领域,纳米载体由于能够减少药物毒性、提高靶向性、增加药物有效性,已经受到越来越多的关注。刺激响应纳米载体是指在光、pH、温度、磁场、氧化剂、还原剂、酶等刺激下,聚合物结构发生改变,从而实现药物可控释放的纳米载体。最近的研究表明,炎症细胞^[2]^、肿瘤细胞^[3]^等病理性细胞会产生过量的活性氧(reactive oxygen species, ROS),包括超氧阴离子( $O_{2}^{\cdot -}$ )、过氧化氢(H_2_O_2_)、羟自由基(^·^OH)、单重态氧(^1^O_2_)等^[4]^。由于ROS具有内源性、高反应活性的特点,因此基于ROS的刺激响应纳米载体成为近年来的研究热点。

ROS响应纳米载体的核心是其骨架聚合物上的ROS响应基团(又称为ROS响应连接子),响应基团对ROS响应,使得聚合物链段发生断裂或者极性变化,进而调控纳米载体释放药物。如表1所示,响应基团可以根据元素的种类,划分为硫族元素类响应基团和其他元素类响应基团。硫族元素类响应基团主要包括硫醚、缩硫酮、硒化物、二硒化物、碲化物,其他元素类响应基团主要包括芳香硼酸酯、过氧草酸酯、二茂铁。

**表1:** ROS响应基团的机理

Element	ROS-responsive linker	ROS-responsive mechanism
Chalcogen	thioether	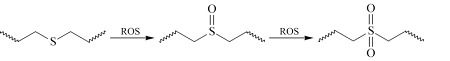
	thioketal	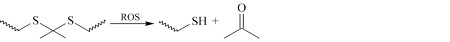
	selenide	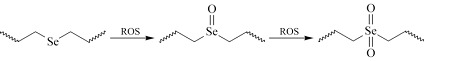
	diselenide	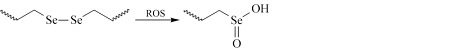
	telluride	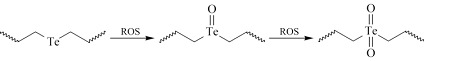
Others	arylboronic ester	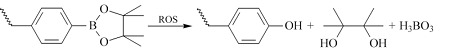
	ferrocene	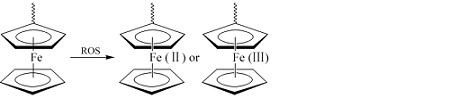
	peroxalate ester	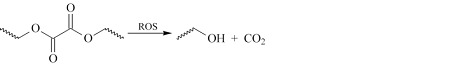

## 1 硫族元素类ROS响应载体

硫醚和缩硫酮都属于硫元素的连接子,但各自的响应机理存在差异。硫醚的响应机理是ROS引起聚合物链段疏水-亲水相变,从而释放药物;而缩硫酮的响应机理是ROS引起聚合物链段断裂,进而释放药物。Cheng等^[5]^使用疏水的苯硫醚基团(PhS)修饰介孔二氧化硅(MSNs)的纳米孔,在ROS响应下,疏水的苯硫醚被氧化成亲水的苯亚砜或苯砜,从而使纳米孔被润湿,导致内部药物的释放;在其研究中,装载罗丹明6G的纳米颗粒MSNs-PhS (1∶20)在100 μmol/L H_2_O_2_中10 h约释放25%;在ROS过表达的MCF-7细胞中明显观察到胞内ROS促使纳米颗粒内部阿霉素释放,而正常HUVEC细胞中只观察到极少量的阿霉素释放。

近几年来,硫族元素中缩硫酮是研究最为广泛的ROS响应载体材料之一。Li等^[6]^利用缩硫酮、美国食品药品监督管理局批准的聚乳酸-羟基乙酸共聚物(PLGA)和聚乙二醇(PEG),以及靶向肽(RGD),合成了聚合物RGD-PEG-TK-PLGA, RGD靶向肿瘤细胞表面的整合素a_v_β_3_,在细胞内ROS刺激下,缩硫酮发生断裂进而使得载体材料释放药物;装载阿霉素的NPs在100 μmol/L KO_2_环境下,6 h约释放58%;对于Cal27细胞,修饰RGD的纳米颗粒的细胞摄取量是未修饰的纳米颗粒的3倍,说明RGD肿瘤靶向性提高了细胞摄取量;小鼠活体实验表明:纳米颗粒(NPs)降低了药物阿霉素的毒性,增加了肿瘤积累,混合装载能刺激细胞产生ROS的*α*-维生素E琥珀酸酯,能够加速缩硫酮断裂释放阿霉素,并且进一步提高其抗肿瘤效果。为了实现协同治疗肿瘤,Chen等^[7]^将缩硫酮引入聚氨基酯骨架中,进一步在载体表面覆盖亲水的藻酸双酯钠,并同时装载光敏剂(IR780)和阿霉素,构建出纳米颗粒PPID;在808 nm激光照射下,IR780引起细胞内温度上升以及ROS大量产生,在ROS响应下,缩硫酮断裂释放阿霉素,实现了光热疗法、光动力疗法、化学疗法的组合;在100 μmol/L H_2_O_2_下,PPID纳米颗粒在20 h释放约40%阿霉素;在Hep1-6细胞中,没有激光照射下,PPID纳米颗粒的半抑制浓度(50% inhibiting concentration, IC50)为0.72 μg/mL阿霉素;在808 nm激光照射下,结合光热疗法、光动力疗法、化疗,肿瘤细胞杀伤效果尤为显著,对于肿瘤治疗具有良好的应用前景。

有研究^[8,9]^表明,线粒体功能紊乱与癌症、神经性疾病等多种疾病有牵连。为了同时解决药物的ROS响应释放和线粒体靶向问题,Zhang等^[10]^直接将缩硫酮与药物喜树碱共价连接,利用靶向肽(cRGD)和三苯基磷(TPP),结合聚二甲基丙烯酰胺,合成了肿瘤细胞和线粒体双重靶向的纳米反应器DT-PNs,实现了肿瘤组织、亚细胞器ROS响应的靶向释放;将细胞靶向的共聚物和线粒体靶向的共聚物共混自组装,cRGD靶向肿瘤细胞表面的整合素a_v_β_3_, TPP靶向线粒体外膜,在线粒体ROS存在下,缩硫酮断裂释放喜树碱,喜树碱引起线粒体ROS产生,促进喜树碱释放,实现自循环;在100 μmol/L H_2_O_2_中80 h释放28%喜树碱;进行肿瘤小鼠实验,DT-PNs明显抑制小鼠肿瘤生长,展现了极好的肿瘤治疗效果。该研究实现了药物的双重靶向和ROS响应释放,在肿瘤细胞线粒体特异性释放喜树碱,并自循环实现ROS爆发和喜树碱大量释放,对于杀伤肿瘤具有良好的效果。

对于硫醚和缩硫酮的ROS敏感性差异,Xu等^[11]^利用硫醚、缩硫酮合成了3种两亲性嵌段共聚物(只含硫醚、只含缩硫酮、同时含硫醚和缩硫酮),比较了3种载体的ROS响应释放情况以及抗肿瘤效果;其中装载阿霉素的只含硫醚的纳米颗粒在500 μmol/L H_2_O_2_中展现出最快的ROS响应释放(72 h释放约65%),对于HeLa细胞和4T1细胞也呈现出最强的肿瘤杀伤效果(IC50分别为0.46和1.29 μg/mL),是很有前景的ROS响应载体。

跟硫同族的硒和碲,在ROS响应方面也引起了关注。硒化物和碲化物性质相似,都是在ROS响应下,疏水的硒化物/碲化物转变成亲水的亚砜/砜,聚合物相变引起内部药物释放;而二硒化物连接子的性质略微独特,具有氧化还原双重响应,氧化环境下二硒键断裂形成硒酸,还原环境下二硒键形成硒醇,断裂引起内部药物的释放。Ma等^[12]^将硒化物引入疏水的聚氨酯嵌段,搭配PEG合成两亲性嵌段共聚物PEG-PUSe-PEG,在ROS响应下,硒化物的疏水-亲水相变使载体发生膨胀、崩解,释放出内部装载的阿霉素;在0.1% H_2_O_2_下10 h释放72%阿霉素,响应释放效果明显优于嵌段共聚物PEG-PUS-PEG(10 h释放41%),初步推测可能是因为元素硒和硫的氧化敏感性差异导致的。该团队^[13]^对此聚合物结构进行了持续并深入的探索,继续引入二硒化物形成嵌段共聚物PEG-PUSeSe-PEG,在0.01% H_2_O_2_或0.01 mg/mL谷胱甘肽(GSH)中都观察到良好的响应以释放罗丹明B。Cao等^[14]^引入碲化物形成嵌段共聚物PEG-PUTe-PEG, ROS响应下发生碲化物的疏水-亲水相变,通过循环伏安法比较含硫、硒、碲的模型化合物的氧化峰,碲化物更低的氧化电位显示出它极好的氧化敏感性,能够对更低浓度的ROS响应,因此碲化物连接子被评价为超敏ROS响应材料。在之前工作的基础上,该团队^[15]^对该两亲性嵌段共聚物进行了改进,用*β*-硒化羰基代替*α*-硒化部分,合成了聚合物C6-C3Se-PEG2000,在ROS响应下,硒化物被氧化为硒亚砜,然后发生硒亚砜分子内消除反应,实现了氧化引起聚合物结构解聚;合成的C6-C3Se-PEG2000能对1 mmol/L H_2_O_2_氧化响应断裂,而超敏碲化物的类似结构正在探索,初步通过^1^H NMR能够观察到C6-C3Te能够对生理条件下(50、100 μmol/L)氧化环境进行响应。

二硒化物由于氧化还原双重响应的特性,能够对肿瘤细胞内的高含量ROS和GSH响应,快速进行药物释放。近年来关于二硒化物连接子的报道较多,Fan等^[16]^利用硒代胱胺、美国食品药品监督管理局批准的PLGA和PEG,合成了ROS响应的载体VPSeP,装载黄连素;在炎症部位,ROS引起二硒键断裂,释放黄连素,黄连素促进ROS生成,进一步激发载体裂解;在10 mmol/L H_2_O_2_中,30 h约释放80%黄连素;在关节炎小鼠实验中,它能够抑制炎症因子IL-1和IL-6的分泌,保护骨关节不被破坏,减轻爪水肿。除了将二硒键引入聚合物结构的研究,Shao等^[17]^直接将含二硒键的有机二氧化硅模块掺入介孔二氧化硅(MSN)中,通过静电相互作用装载核糖核酸酶A,进一步包裹HeLa细胞的膜碎片,成功合成了具有同源靶向性、氧化还原双重响应的纳米颗粒;此时,氧化还原响应更为灵敏的MSN2纳米颗粒在100 μmol/L H_2_O_2_中10 h约释放55%核糖核酸酶A,在5 mmol/L GSH中10 h约释放50%核糖核酸酶A;将纳米颗粒分别与HeLa细胞、MCF-7细胞共孵育,在HeLa细胞中观察到更高的荧光强度,说明了载体同源靶向性;在肿瘤小鼠实验中,也明显观察到了肿瘤生长抑制。

## 2 其他元素类ROS响应载体

芳香硼酸酯作为ROS响应连接子,能够被氧化成苯酚和硼酸,在ROS响应载体方面也获得了很大的关注。Broaders等^[18]^利用芳香硼酸酯作为连接子,合成了氧化敏感的纳米颗粒Oxi-DEX,即用芳香硼酸酯修饰右旋糖苷的羟基,使聚合物链段由水溶性转变为油溶性,进而实现模型抗原-鸡卵白蛋白(OVA)的包载;在H_2_O_2_环境下,芳香硼酸酯降解,暴露出右旋糖苷的羟基,最终使得聚合物链段转换为原始的水溶状态,即使聚合物链段发生极性变化,释放出OVA;在1 mmol/L H_2_O_2_下,2 h后Oxi-DEX发生完全的相变;装载OVA的Oxi-DEX明显引起DC2.4小鼠神经系统树突细胞的MHC Ⅰ抗原表达增强,该载体能够作为快速的选择性给药系统。另外,有报道表明,树突细胞的吞噬体内ROS浓度能够高达1 mmol/L^[19]^, Oxi-DEX能够实现生理环境下的ROS响应,但是对于ROS浓度低至100 μmol/L的肿瘤细胞,它并不能实现响应。为了解决这一问题,De Gracia Lux等^[20]^利用芳香硼酸酯合成了两种聚合物,聚合物1是芳香硼酸酯直接连接主链,聚合物2是芳香硼酸酯通过苄基醚连接主链,聚合物1形成的载体1在1 mmol/L H_2_O_2_中26 h约释放50%尼罗红,而聚合物2却能在100 μmol/L H_2_O_2_中达到类似释放效果;将载体2装载荧光素二乙酸,与中性粒细胞孵育,佛波酯处理6 h后,通过荧光强度观察到释放增加了2倍,使得芳香硼酸酯纳米载体能够在肿瘤细胞生理环境下ROS响应释放。

Deng等^[21]^利用芳香硼酸酯形成了4种H_2_O_2_响应的单体,选择单体NBMA形成两亲性嵌段共聚物,利用线粒体靶向肽(CGKRK)进行表面功能化,合成了具有线粒体靶向、氧化响应的聚合物囊泡;在线粒体H_2_O_2_响应下,聚合物发生级联消除和脱羧反应,疏水性双分子层发生酰胺反应,导致聚合物囊泡内部交联,使得双分子层发生疏水-亲水相变,从而释放出疏水性双分子层封装的紫杉醇,以及水性内腔封装的盐酸阿霉素;载体Gd-N8在1 mmol/L H_2_O_2_中24 h约释放94%盐酸阿霉素和93%紫杉醇;将小分子细胞核染料(DAPI)和大分子的右旋糖苷共同封装在亲水内腔,与佛波酯处理后的HeLa细胞共孵育12 h,观察到大部分的蓝色荧光DAPI从囊泡扩散进入细胞核,而红色荧光的右旋糖苷与囊泡出现极好的共定位,说明氧化环境促使疏水性双分子层发生相变,小分子DAPI发生渗透,双分子层交联保持了囊泡结构的完整性,大尺寸的右旋糖苷被保留在囊泡内腔。该载体实现了两种物理性质不同的药物同时封装,并且通过ROS响应促使囊泡的双分子层发生相变,在保持了囊泡结构完整性的基础上,实现了药物的释放。

芳香硼酸酯形成的载体能够对生理相关的H_2_O_2_浓度响应,获得了较好的发展。而含二茂铁的聚合物是将金属引入聚合物,虽然由于优异的物理性质获得了关注,但是在ROS响应方面,仍需要进一步的发展。Na等^[22]^合成了一系列含二茂铁的两亲性嵌段共聚物FMMA-r-MA,在水中自组装形成内核疏水的纳米颗粒,氧化后疏水的二茂铁分子变成亲水的二茂铁阳离子,发生疏水-亲水相变,同时由于阳离子的静电排斥作用会让纳米颗粒膨胀破碎,释放内部的尼罗红,氧化响应和稳定性最好的FNP (C2)纳米颗粒在0.4 mol/L H_2_O_2_环境下,8 h约释放25%尼罗红。

与Li等^[6]^和Fan等^[16]^设计的结构类似,Liang等^[23]^使用过氧草酸酯连接PLGA和PEG,合成聚合物3s-PLGA-PO-PEG,装载模型抗原OVA,在ROS响应下,过氧草酸酯断裂释放OVA,使用聚醚酰亚胺修饰纳米颗粒表面以增加转染效率,从而构建出PPO纳米颗粒;PPO纳米颗粒在200 μmol/L H_2_O_2_中两天释放超过90%OVA;在小鼠活体实验中,PPO纳米颗粒能够引起OVA特异性抗体的生成,从而上调CD4^+^和CD8^+^T细胞的比例,激活记忆T细胞。这种携带抗原疫苗的NPs能够实现体内的免疫响应。

如表2所示,对于ROS刺激响应纳米载体,缩硫酮和芳香硼酸酯作为ROS响应基团,引入纳米载体较为广泛,而且ROS响应也较为灵敏,两者在细胞层面皆有良好的应用。二硒化物不仅是ROS响应基团,而且是GSH响应基团,对于ROS和GSH都呈现高含量的肿瘤细胞,二硒化物的双重响应能够促使药物的响应释放;但是由于细胞质内也呈现高浓度的GSH,对于亚细胞器的特定响应释放,二硒化物还呈现一定的劣势。结构和性质十分类似的硫醚、硒化物和碲化物中,目前硒化物和碲化物的ROS响应载体发展较少,可能是出于它们的生物层面安全性考虑,或者是载体制备的困难程度影响;但是由于碲化物的ROS超敏能力,它还具有较大的应用潜力。ROS响应基团中的过氧草酸酯和二茂铁在ROS响应方面还需要进一步的发展,以获得生理条件下的响应。

**表2:** ROS刺激响应纳米载体的性能比较

ROS- responsive linker	Responsive mechanism	Nanocarriers	Diameter/ nm	Drug	ROS-responsive release (in vitro)	Cell type	Reference
Thioether	hydrophobic/ hydrophilic conversion	MSNs-PhS (1∶20)	319	Rhodamine 6G/ doxorubicin	+++	MCF-7	[2]
Thioketal	cleavage	RGD-PEG-TK-PLGA	115	doxorubicin/α-TOS	++++	Cal27	[3]
		PPID	198	IR780/doxorubicin	+++	Hep1-6	[4]
		DT-PNs	55	camptothecin	++	4T1	[5]
Selenide	hydrophobic/ hydrophilic conversion	PEG-PUSe-PEG	71	doxorubicin	++	/	[7]
	selenoxide elimination reactions	C6-C3SePEG2000	84	/	/	/	[10]
Diselenide	cleavage	PEG-PUSeSe-PEG	76	Rhodamine B	++++	/	[8]
		VPSeP	153	berberine	++	HFLS-RA	[11]
		MSN2	50	Ribonuclease A	++++	HeLa	[12]
Telluride	hydrophobic/ hydrophilic conversion	PEG-PUTe-PEG	35	/	/	/	[9]
Arylboronic	cleavage	Oxi-DEX	100	ovalbumin	/	DC 2.4	[13]
ester		Polymer 2	136	Nile Red/fluorescein diacetate	+++	Neutrophils	[14]
		Gd-N8	490	paclitaxel/doxorubicin hydrochloride	++++	Hela	[15]
Ferrocene	hydrophobic/ hydrophilic conversion	FNP (C2)	190	Nile red	+	/	[16]
Peroxalate ester	cleavage	PPO	220.4±1.8	ovalbumin	+++	BMDC	[17]

MSNs-PhS: mesoporous silica nanocarriers modified with phenyl sulfide groups; RGD-PEG-TK-PLGA: arginine-glycine-aspartic acid sequences containing peptides-polyethylene glycol-thioketal-poly(lactic-co-glycolic acid); PPID: a propylene glycol alginate sodium sulfate-coating nanoparticle composed of poly(*β*-amino ester), IR780 and doxorubicin; DT-PNs: cancer cell and mitochondria dual-targeting polyprodrug nanoreactors; PEG-PUSe-PEG: an amphiphilic block copolymer with a hydrophobic selenide-containing polyurethane blocks and two hydrophilic poly(ethylene glycol) blocks; C6-C3Se-PEG2000: an amphiphilic block copolymer composed of bis (6-hydroxyhexyl) 3,3'-selenodipropanoate, 2,4-toluenediisocyanate and poly(ethylene glycol) monomethylether; PEG-PUSeSe-PEG: an amphiphilic block copolymer with a hydrophobic diselenide-containing polyurethane blocks and two hydrophilic poly(ethylene glycol) blocks; VPSeP: vitamin E succinate-poly (lactic-co-glycolic acid)-selenocystamine dihydrochloride-methoxy poly(ethylene glycol) co-polymers; MSN2: a cancer cell membrane-coating mesoporous silica nanoparticles composed of diselenide-bond-containing organosilica moieties; PEG-PUTe-PEG: an amphiphilic block copolymer with a hydrophobic telluride-containing polyurethane blocks and two hydrophilic poly(ethylene glycol) blocks; Oxi-DEX: oxidation-sensitive dextran carrier microparticles; Polymer 2: a polymer with an ether linkage between the boronic ester group and the polymeric backbone; Gd-N8: a MR imaging contrast agent-conjugating block polymer composed of monomer NBMA and Poly(ethylene oxide) monomethyl ether; FNP(C2): a ferrocene-containing polymers with ferrocenylmethyl methacrylate and methacrylic acid monomers(0.4:2, molar ratios); PPO: a Poly(ethylene imine)-containing polymer with a peroxalate ester bond between poly(lactic-co-glycolic acid) and poly(ethylene glycol); IR780: 2-[2-[2-chloro-3-[(1,3-dihydro-3,3-dimethyl-1-propyl-2h-indol-2-ylidene) ethylidene]-1-cyclohexen-1-yl]ethenyl]-3,3-dimethyl-1-propylindolium iodide; /: no information. The amount of “+” quantifies the ROS-responsive release of nanocarriers.

## 3 总结

由于疾病部位的ROS水平异常,因此ROS响应纳米载体具有靶向给药、降低药物毒性等优点,具有广阔的应用前景和发展潜力。制备ROS响应纳米载体时,ROS响应基团的氧化敏感性值得考虑,本文中的硫醚、硒化物、碲化物、二茂铁都是ROS引起其疏水-亲水相变,进而引起药物释放,特别是对于硒化物,*β*-硒化羰基代替*α*-硒化部分后,硒化物被氧化为硒亚砜,会继续发生硒亚砜消除反应,引起聚合物断裂;缩硫酮、二硒化物、芳香硼酸酯、过氧草酸酯都是ROS引起其断裂进而释放。ROS响应纳米载体用于活体的研究才刚刚起步,载体的生物相容性、生物降解性尤其需要关注,只有在确保安全的前提下,ROS响应纳米载体才能起到对疾病部位进行智能释放药物、精准治疗的目的。
